# Analysis of Tissue Repair of a New Cement Based on Calcium and Strontium Aluminates: A Triple-Blinded, Randomized, Controlled Trial in an Animal Model

**DOI:** 10.1055/s-0044-1786875

**Published:** 2024-05-22

**Authors:** Elizandra Silva da Penha, Nonato Amorim de Farias Filho, Luanna Abílio Diniz Melquíades de Medeiros, Rosana Araújo Rosendo, Marco Antônio Dias da Silva, Willams Teles Barbosa, Raúl García-Carrodeguas, Miguel Angel Rodríguez, Eliseu Aldrighi Münchow, Rogério Lacerda-Santos, Marcus Vinícius Lia Fook

**Affiliations:** 1 Department of Dentistry, Dental School, Federal University of Campina Grande, Patos, Paraíba, Brazil; 2 SENAI Institute of Innovation (ISI) in Forming and Joining (CIMATEC ISI F&J), Salvador, Bahia, Brazil; 3 Noricum S.L., San Sebastian De Los Reyes, Madrid, Spain; 4 Institute of Ceramics and Glass (ICV-CSIC), Cantoblanco, Madrid, Spain; 5 Department of Conservative Dentistry, Federal University of Rio Grande do Sul, Porto Alegre, Rio Grande do Sul, Brazil; 6 Department of Orthodontics, Federal University of Juiz de Fora, Governador Valadares, Minas Gerais, Brazil; 7 Department of Materials Engineering, Federal University of Campina Grande, Campina Grande, Paraíba, Brazil

**Keywords:** microscope, biocompatibility, histology, root canal filling sealer

## Abstract

**Objective** The focus of this triple-blind randomized study was to evaluate the biocompatibility of a new root canal filling sealer (RCFS) based on tristrontium aluminate and dodecacalcium hepta-aluminate in living tissue.

**Material and Methods** Forty-five Wistar rats ( *Rattus norvegicus* ) were divided into three groups: control (polyethylene), sealer (Bio-C Sealer, Londrina, PR, Brazil), and experimental (tristrontium aluminate and dodecacalcium hepta-aluminate). The tissues were analyzed under an optical microscope to assess different cellular events at different time intervals (7, 15, and 30 days).

**Statistical Analysis** Data were analyzed using the Kruskal–Wallis and Dunn ( *p* < 0.05) tests.

**Results** In the initial period, a moderate inflammatory infiltrate was observed, similar between the endodontic cements groups ( *p* = 0.725). The intensity of the infiltrate decreased with time, with no significant difference among the groups ( *p* > 0.05). The number of young fibroblasts was elevated in all groups evaluated at 7 days. The experimental group showed the highest number of cells at all time intervals, but the difference with the sealer group at 7 ( *p* = 0.001) and 15 days ( *p* = 0.002) and the control group at 30 days was not significant ( *p* = 0.001). Regarding tissue repair events, the amount of collagen fibers increased over the experimental intervals, with no significant difference between the sealer and control groups ( *p* > 0.05).

**Conclusion** The experimental RCFS based on calcium and strontium aluminates proved to be biocompatible for use in close contact with periapical tissue, inducing a low inflammatory reaction and favoring rapid tissue repair.

## Introduction

The root canal filling sealer (RCFS) is an important component of the filling process because it provides adhesion of gutta-percha to the dentinal walls. 1 These RCFSs must meet certain basic requirements to ensure safety and effectiveness, such as biocompatibility, 1 2 3 4 ability to seal the root canal, radiopacity, dimensional stability, and microbicidal and bactericidal properties. 1 2

Mineral trioxide aggregate (MTA) is one of the best known RCFSs. 5 Its composition is approximately 80% by weight of Portland sealant and 20% by weight of bismuth oxide (Bi _2_ O _3_ ), included as a radiopacifying agent. However, Bi _2_ O _3_ has been shown to interfere with hydration kinetics, reducing the precipitation of calcium hydroxide in the hydrated paste, 6 affecting the compressive strength 7 and delaying hardening. 5 In addition, the toxicity due to the Bi _2_ O _3_ compounds themselves and the associated arsenic impurities have raised concerns about the safety of MTA. 8 RCFSs based on calcium aluminate represent a promising alternative, exhibiting physicochemical characteristics such as a thermal coefficient and chemical composition similar to human teeth and bone, controllable rheology and short setting time at room temperature, high initial strength, 9 and bioactivity. 10 In particular, dodecacalcium hepta-aluminate has rapid hydration, and its configuration can be controlled by adding additives to the mixture. 11

In this line, strontium aluminates have also shown potential as RCFSs. 12 They can provide higher X-ray absorption and radiopacity than their Ca aluminate counterparts and have shown the ability to significantly inhibit bone resorption and improve bone regeneration in animals. 13 14 Tristrontium aluminate is the most hydraulic of this class of compound, as when mixed with water, it becomes an RCFS paste with short handling and setting times. Hydration begins immediately and progresses rapidly without any induction period but with considerable production of heat. 12

The combination of tristrontium aluminate and dodecacalcium hepta-aluminate has demonstrated higher hydration rates, a shorter setting time that can better adapt to the time span of clinical interventions, and an intense bioactive response. 15 However, no study has been performed to evaluate the *in vivo* biocompatibility of this new RCFS. Thus, this study aimed to evaluate the biocompatibility of RCFSs based on tristrontium aluminate and dodecacalcium hepta-aluminate in living tissue.

## Materials and Methods

### Animal Model and Experimental Groups

The study was approved by the Committee for Ethics in Animal Research, protocol CEP/CEUA/CSTR No 032023. The sample size calculation was based on a pilot study. For a standard deviation of 0.44 and a minimal intergroup difference of 1 for the inflammatory infiltrate to be detected, a sample of five animals is required to provide a statistical power of 80% with an alpha of 0.05. Forty-five male Wistar rats ( *Rattus norvegicus* ) weighing 200 to 250 g and aged between 3 and 4 months were obtained from the vivarium of the Center for Education and Health of the Federal University of Campina Grande (UFCG). The animals were housed in the INSIGHT vivarium cabinet of the Materials Bioassay Laboratory (LBio) of the UFCG, with a controlled temperature of 23 ± 1°C and ambient humidity of 41%, and maintained on chow and water *ad libitum* . 16 17 18

**Fig. 1:**
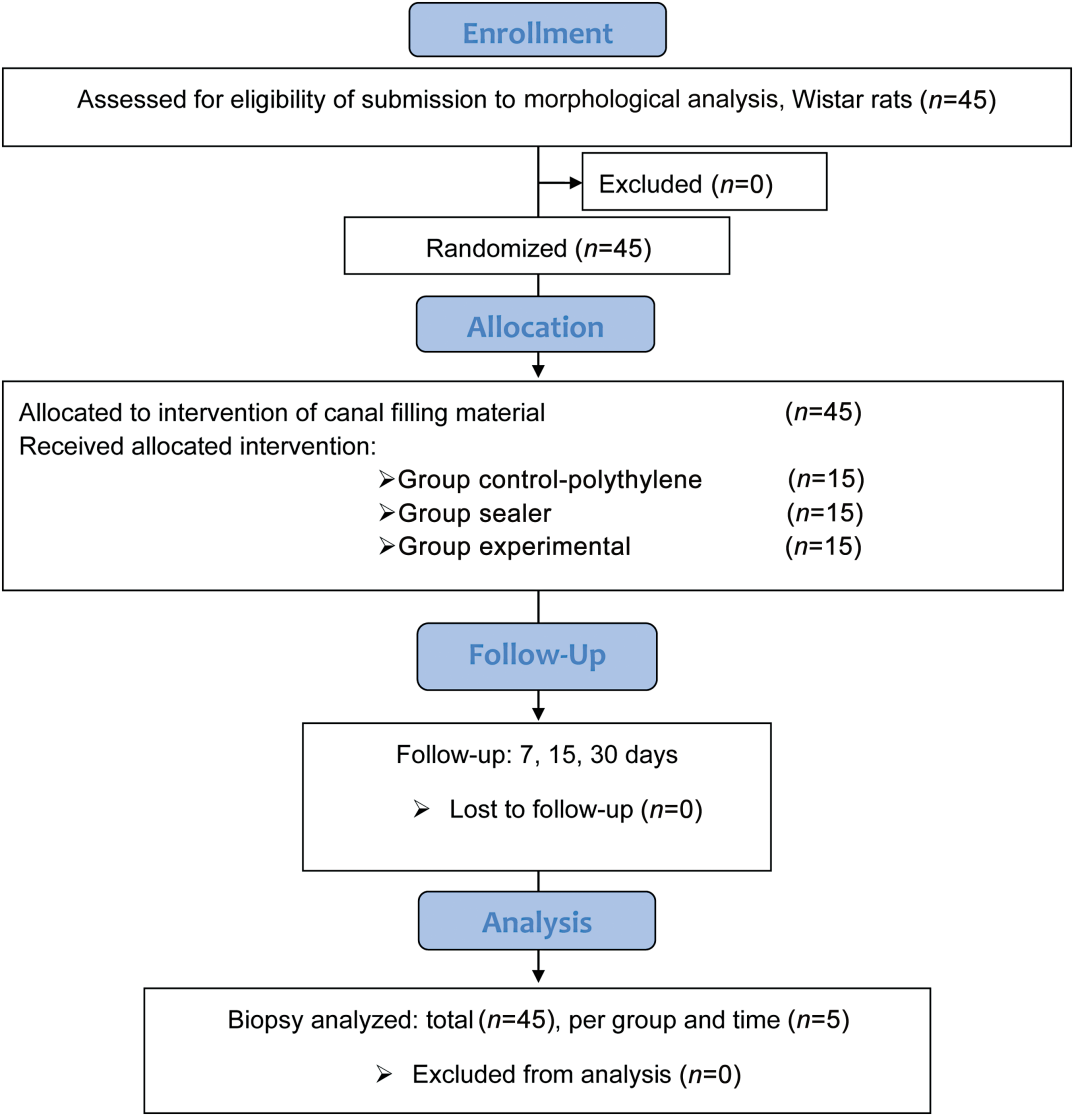
Flow diagram of the included animals and experimental groups.

The animals were randomly distributed into three groups according to the material to be implanted: the Sealer group (Bio-C Sealer bioceramic filling sealant, Lot: 66041, Angelus S.A., Londrina, PR, Brazil), the experimental group (filling sealant based on tristrontium aluminate and dodecacalcium hepta-aluminate) according to Barbosa et al 15 who evaluated the physicochemical and bioactivity properties of this compound previously, and the control group (polyethylene tube), which were implanted with a biologically inert tube without the presence of filling sealant ( Fig. 1 ). Previously, the tubes were autoclaved at a temperature of 120° C for 20 minutes and then used as inoculation vehicles for the tested materials. 19 20

Each specimen was implanted in the subcutaneous tissue on the back of the animal bilaterally. The side of the biopsy to be used was defined randomly, using the closed envelope process; chosen, and opened by an examiner external to the study. In the absence of material implanted in the biopsy on the side of choice, the opposite side was used. The three groups were further distributed into three sampling times: 7, 15, and 30 days, for a total of nine groups. 21 22

### Manipulation of Cements

The experimental filling material underwent formulation and characterization based on the tests performed by Barbosa et al. 15 The resulting cement consisted of a paste composed of tristrontium aluminate (80%) and dodecacalcium hepta-aluminate (20%) mixed with aqueous polyethylene glycol (30%), with a powder to liquid ratio equal to 0.39 mL/g. The filling material showed working characteristics suitable for clinical application, with a setting time of 60 minutes without excessive heat development, radiopacity equivalent to 3.1 mm of Al, and compressive strength of 5 MPa in 24 hours after sufficient mixing for use in places with low mechanical stress. 15 After 24 hours of preparation of the powder and liquid, they were manipulated as recommended previously. 15

The filling material was introduced into the openings at the extremities of the tubes using a syringe (Centrix, Connecticut, United States) supported on a glass slide at one end and a small glass slide at the other to flatten the material. 23 24 25 Each cement was placed in a polyethylene tube 10 mm in length and 1.5 mm in diameter, with the exception of the tubes used in the control group, which were implanted and left empty. 24 The sealer cement, based on calcium silicate, was manipulated according to the manufacturer's specifications.

### Surgical Procedure

For the surgical procedure, anesthesia was performed with intraperitoneal injections of ketamine hydrochloride (0.10 mL/kg, Vetnil, Vetecia, SP, Brazil), 2% xylazine hydrochloride (0.1 mL/kg, União Química, SP, Brazil), and 0.9% sodium chloride (0.15 mL/kg, Fresenius Kabi, Barueri, SP, Brazil). 18 Antisepsis with 2% chlorhexidine solution (Riohex, Rioquímica, SP, Brazil) was used for trichotomy of the bilateral scapular region to an anteroposterior length of 2 cm. A 1-cm-long incision was made with a No. 15 scalpel blade for implantation of the material for each group. 26

With hemostatic forceps, the tissues were divided laterally, allowing insertion of the tube into the surgical site. To facilitate localization of the area of histological interest for the study, all tubes were implanted to the right of the incision and with a single opening in the cranial direction. The surgical edges were closed by simple three-point suture using 3.0 silk thread (Ethicon, Somerville, New Jersey, United States). After the surgical procedure, two drops of sodium dipyrone (500 mg/mL, Neo Química, Anápolis, GO, Brazil) were administered orally immediately after suturing was completed. In the postoperative period, the rats were returned to the animal house, where they were kept under daily observation until they underwent biopsy at 7, 15, and 30 days.

All procedures in this study were performed in accordance with the guidelines of the *Canadian Council on Animal Care* . 27 After the sampling periods, the animals were sedated with ketamine hydrochloride (0.10 mL/kg, Vetnil, Vetecia, SP, Brazil), 2% xylazine hydrochloride (0.1 mL/kg, União Química, SP, Brazil), and 0.9% sodium chloride (0.15 mL/kg, Fresenius Kabi, Barueri, SP, Brazil) and sacrificed by cervical dislocation. 24 28 Euthanasia was confirmed by observing the absence of the eyelid reflex, chest movements, and heartbeat for 3 minutes.

Next, the implanted specimen underwent tissue biopsy along with a safety margin of adjacent connective tissue of 5 mm. 29 The samples were stored in individual, labeled containers and fixed in 10% buffered formalin solution (pH 7.4) for 7 days.

### Morphological Analysis—Biocompatibility

After fixation, the biopsy samples were prepared for histological processing, and 6-µm- thick serial sections were obtained and stained with hematoxylin (Proquimios, Rio de Janeiro, RJ, Brazil) and eosin (Dinâmica, Química Contemporânea LTDA, Indaiatuba, SP, Brazil) (HE) for evaluation under optical microscopy. 30

All histological slides were photodocumented at a standardized magnification of 400x with a Leica DM500 binocular optical microscope (Leica-Microsystems, Wetzlar, Germany) using the rear digital camera of a smartphone (iPhone 10, Apple, Cupertino, California, United States) attached to the microscope through an adapter custom built for this function. For each sample, five sections representative of the histological condition of the tissue adjacent to the implanted cements were analyzed 19 20 as well as five equal and equidistant areas in the tissue surrounding the implanted specimen. The microscopic evaluation in this analysis was performed by a single calibrated researcher ( *κ* = 0.85).

For the cellular variables, including the number of fibroblasts, fibrocytes, multinucleated giant cells, and blood vessels in each area, 31 counts were performed using ImageJ version 1.51 (National Institute of Health, United States) by using its 10 × 10 mesh tool to create a field containing 100 equidistant points, where only cells located in these intersections were counted. 32 The values obtained in each of these fields were added together, thus establishing the total number of cells; subsequently, these data were used to calculate the average value/per field of each group. The evaluator was previously calibrated ( *κ* = 0.85) in a blinded evaluation. 31

For the cellular variables inflammatory infiltrate, edema, necrosis, granulation tissue, and collagen, the five sections representative of the histological condition of the tissue were histologically evaluated according to the following scores: 0—absent (when absent in the tissue); 1—sparse (when little was present, or in very small groups), 2—moderate (when densely present, or in a few groups), and 3—intense (when found throughout the field, or present in large numbers). 19 20 These values represent the mean scores of the sum of five histological sections representative of the evaluated tissue ( *n* = 5, per group). The histological sections were evaluated randomly at five different areas of the tissue adjacent to the specimen. The microscopic evaluation in this analysis was performed by a single calibrated researcher ( *κ* = 0.90).

This study was randomized and triple-blind; all experimental materials used in the animals were placed in groups I to III, so that the examiner and the statistical evaluator were not aware of the materials used.

### Statistical Analysis

The distribution of the data was analyzed by the Kolmogorov‒Smirnov test (Graph Pad-Prism 5.0, San Diego, California, United States). The results for the cellular events did not present a normal distribution; therefore, they were subjected to the Kruskal‒Wallis and Dunn test. *p-* Value less than 0.05 was considered statistically significant.

## Results

**Fig. 2:**
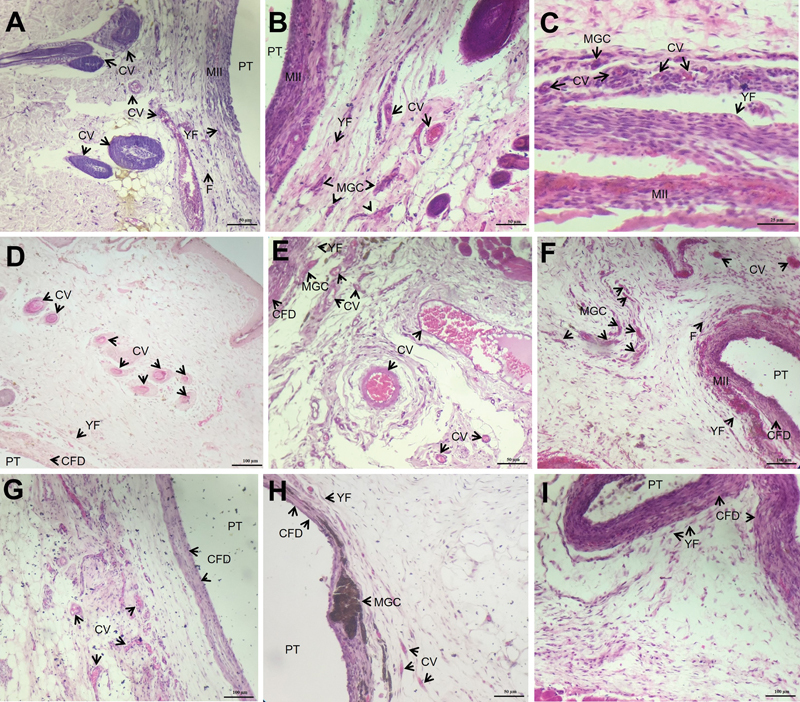
Photomicrographs of the histological samples. ( **A** ) Seven days after implantation, control group: mild inflammatory infiltrate (MII), presence of congested blood vessels (CV), fibrocytes (F) and young fibroblasts (YF) around the polyethylene tube (PT) (HE, 200× magnification, scale: 50 μm). ( **B** ) Seven days after implantation, Group Sealer: moderate inflammatory infiltrate (MII), expressive presence of multinucleated giant cells (MGC), congested blood vessels (CV), and young fibroblasts (YF) around the polyethylene tube (PT) (HE, 200× magnification, scale: 50 μm). ( **C** ) Seven days after implantation, experimental group: moderate inflammatory infiltrate (MII), presence of multinucleated giant cells (MGC), congested blood vessels (CV) and young fibroblasts (YF) (HE, 400× magnification, scale: 25 μm). ( **D** ) Fifteen days after implantation, Group Control: presence of congested blood vessels (CV), young fibroblasts (YF) and collagen fiber deposition (CFD) around the polyethylene tube (PT) (HE, 100× magnification, scale: 100 μm). ( **E** ) Fifteen days after implantation, Group Sealer: presence of congested blood vessels (CV), multinucleated giant cells (MGC), young fibroblasts (YF) and onset of collagen fiber deposition (CFD) (HE, 200× magnification, scale: 50 μm). ( **F** ) Fifteen days after implantation, experimental group: mild inflammatory infiltrate (MII), congested blood vessels (CV), expressive presence of multinucleated giant cells (MGC), fibrocytes (F) and young fibroblasts (YF), onset of collagen fiber deposition (CFD) around the polyethylene tube (PT). (HE, 100× magnification, scale: 100 μm). ( **G** ) Thirty days after implantation, Group Control: presence of congested blood vessels (CV), area of collagen fiber deposition (CFD) disposed in parallel bands, around the polyethylene (PT) tube (HE, 100× magnification, scale: 100 μm). ( **H** ) Thirty days after implantation, Group Sealer: presence of congested blood vessels (CV), expressive response by multinucleated giant cells (MGC), young fibroblasts (YF) and collagen fiber deposition (CFD) disposed in parallel bands, around the polyethylene (PT) tube (HE, 200× magnification, scale: 50 μm). ( **I** ) Thirty days after implantation, Experimental Control: presence of young fibroblasts (YF), area of collagen fiber deposition (CFD) disposed in parallel bands, around the polyethylene (PT) tube (HE, 100× magnification, scale: 100 μm).

In the initial evaluation period, mild inflammatory infiltrate was demonstrated in the control group, while both filling material groups demonstrated moderate inflammatory infiltrate; the difference between them was not significant ( *p* = 0.725; Fig. 2A–C ). The intensity of the inflammatory infiltrate was inversely proportional to the experimental time interval, with no significant difference among groups ( *p* > 0.05; Fig. 2A–C , Table 1 ).

Circulatory changes (edema) were most present at 7 days, but there was a significant difference only between the control group and the sealer and experimental groups at 15 days ( *p* = 0.006). Tissue degeneration (necrosis) and granulation tissue were not substantial and did not show significant differences among the evaluated groups ( *p* > 0.05).

The blood vessel count showed a higher amount at 30 days in the cement groups, especially in the experimental group, which showed an increasing count over the observation intervals. There was a significant difference between the experimental group and the control group at 7 days ( *p* = 0.020; Fig. 2A and C ) and 30 days ( *p* = 0.002) and between the experimental group and the sealer group at 15 days ( *p* = 0.022; Fig. 2D–F , Table 1 ).

Multinucleated giant cells were more abundant in the sealer and experimental groups, and there was a significant difference between the sealer and control groups at 7 days ( *p* = 0.001; Fig. 2A–B ) and 30 days ( *p* = 0.001; Fig. 2G–I ) and between the experimental and control groups ( *p* = 0.001) at 15 days ( Fig. 2D and F , Table 1 ).

All groups evaluated had greater numbers of young fibroblasts in the initial, 7-day period, the result of the initial reaction for tissue repair ( Fig. 2A–C ), followed by a quantitative decrease proportional to the experimental time interval ( Fig. 2D–I ). The experimental group showed the highest cell quantity at all time intervals ( Fig. 2C , 2F and 2I ), with no significant difference from the sealer group at 7 ( *p* = 0.001) and 15 days ( *p* = 0.002) or with the control group at 30 days ( *p* = 0.001) Table 1 .

Fibrocytes were found in greater quantity in the control group at 7 days ( *p* = 0.004) and 30 days ( *p* = 0.115) ( Fig. 2A and G ). The sealer group had fewer fibrocytes at 15 days ( *p* = 0.004) than the experimental group ( Fig. 2E and F ).

Regarding tissue repair events, the amount of collagen fibers increased over the experimental intervals, with no significant difference between the cement groups and the control group ( *p* > 0.05; Fig. 2A–I , Table 1 ).

## Discussion

New materials for clinical use should be initially evaluated by histocompatibility tests, 33 human tissue, 34 cell culture, 35 and implantation in living tissues. 19 36

The healing of damaged tissue is initiated by signals sent by the immune cells present in the inflammatory infiltrate. 37 In this study, an RCFS based on tristrontium aluminate and dodecacalcium hepta-aluminate was compared with a bioceramic RCFS – Sealer Plus BC, which is based on calcium di- and trisilicate and zirconium oxide, to evaluate its tissue response in living tissue.

Empty polyethylene tubes were used as controls in this study, as they are considered biologically inert. The tissue damage caused was the response of the surgical procedure to the implantation of the tubes, with lower inflammatory intensity than in the filling material groups, corroborating other studies. 21 32 38

At 7 days, both RCFSs showed a moderate inflammatory reaction 39 that was similar between the groups ( *p* = 0.725). The intensity of the inflammatory infiltrate was inversely proportional to the experimental time interval, with no significant difference between groups ( *p* > 0.05). Studies 40 41 have suggested that the fixation reaction of RCFSs with similar compositions promotes the formation of calcium and hydroxyl (OH _2_ ) ions, and the alkaline pH 3 40 41 stimulates the recruitment of inflammatory cells and the production of cytokines. In addition, the proinflammatory cytokine interleukin-6 (IL-6), which can activate and modulate specific cells, plays an important role in the inflammatory reaction and bone resorption. 39 42 In RCFSs similar to the one in this study, a significant increase in the amount of IL-6 was observed, greatest at 7 days followed by a gradual decrease. 43

Circulatory changes (edema) were more present at 7 days, but there was a significant difference only between the control group and the sealer and experimental groups at 15 days ( *p* = 0.006). Tissue degeneration (necrosis) and granulation tissue were not substantial and did not show significant differences between the evaluated groups ( *p* > 0.05).

Angiogenesis is based on the formation of new blood vessels and partially driven by inflammation because the creation of new vessels is related to increased nutrition, oxygenation, and a greater number of immune cells 23 25 44 and repair of the damaged area. 45 The blood vessel count was higher at 30 days in the RCFSs, especially in the experimental group, which showed increasing counts over the experimental intervals. There was a significant difference between the experimental group and the sealer group at 15 days ( *p* = 0.022).

Multinucleated giant cells are formed from the union of macrophages 37 for the isolation and elimination of foreign body reactions. 2 The multinucleated giant cells were closer to the sealer particles and were more present in the sealer group at 7 and 15 days ( *p* < 0.05) and in the experimental group at 15 days ( *p* < 0.05). This suggests that the RCFS may have released more irritants 46 in the initial intervals. The presence of RCFS remnants near the cavity observed in some samples in later periods demonstrated that these biomaterials were not easily digested by the multinucleated giant cells or quickly eliminated by local lymphatic drainage. 47

Fibrocytes, which play an important role in the recovery of damaged tissue, are able to differentiate into fibroblasts. 37 These cells were found in greater quantity in the control group at 7 days ( *p* = 0.004), having proliferated due to the increased need for tissue healing. A lower amount of fibrocytes was observed in the sealer group at 15 days ( *p* = 0.004) than in the experimental group, which could indicate later recruitment by the RCFS based on calcium and strontium aluminates than by the others. The increase in these cells at 30 days in the control and sealer groups indicates a decrease in fibroblast differentiation, resulting from the stabilization of the scar tissue.

An important characteristic of cements is their bioactivity, wherein they induce repair in damaged tissues in their surroundings, 3 39 42 stimulate tissue repair, and induce mineralization, promoting an integration of the material with dental tissues. 1 However, this property was not evaluated in this study. Barbosa et al 15 showed the bioactive capacity of an experimental cement formed from apatite after 3 days in simulated body fluid solution.

Young fibroblasts were more numerous in all groups at 7 days, the result of the initial reaction for tissue repair, followed by a quantitative decrease proportional to the experimental time interval. The experimental group showed the highest cell quantity at all time intervals but was not significantly different from the sealer group at 7 ( *p* = 0.001) and 15 ( *p* = 0.002) days. These results corroborate the fact that neither cement caused an intense inflammatory process, since the presence of fibroblasts decreased over time due to the process of tissue remodelling. 48 However, at 30 days, a significant difference in the number of fibroblasts was observed between the cements, which suggests a slower healing response in this period for the experimental cement.

Regarding tissue repair events, the amount of collagen fibers increased over the experimental intervals, with no significant difference between the RCFSs and the control group ( *p* > 0.05). In addition, the presence of well-oriented collagen fibers arranged in bundles in the capsules indicated that the inflammatory reaction was gradually replaced by dense connective tissue. This hypothesis is reinforced by the reduction in the thickness of the capsule at the 30-day evaluation period.

The Sealer Plus has calcium disilicate, nanoparticulate calcium trisilicate, and zirconium oxide in its formulation; the experimental RCFS was formulated with tristrontium aluminate and dodecacalcium hepta-aluminate. These differences may have affected the physicochemical properties as well as the biocompatibility and bioactive potential of the RCFS. 3 39 42

Calcium aluminates have been used as dental and bone fillers, exhibiting controllable rheology and short setting time at room temperature, high initial strength, bioactivity, and biocompatibility. 10 11 They exhibit certain physicochemical characteristics, such as their thermal coefficient and chemical composition, which are similar to those of human teeth and bone. 49 For the strontium aluminates, since the Sr cross-section is larger than that of Ca, it provides greater absorption of X-rays and radiopacity, and they are more radiopaque than their Ca aluminate counterparts. In addition, they significantly inhibit bone resorption and improve bone regeneration in animals with induced osteoporosis. 13

From a clinical view, the experimental RCFS 15 did not cause relevant tissue damage, allowed normal tissue recovery, and proved to be a bioinert biomaterial. 22 This corroborates other authors who showed regression of the inflammatory reaction and scar repair in similar RCFSs. 3 39 42

The results found in this study indicate that this new RCFS 15 has the potential to be applied in root canal treatments. However, for it to be effectively used in clinical procedures, human clinical trials involving endodontic canals should be conducted. 50

## Conclusion

The experimental filling sealant based on calcium aluminates and strontium proved to be biocompatible for use in close contact with periapical tissue, inducing a low inflammatory reaction and favoring rapid tissue repair.

**Table 1:** Mean of the scores a attributed to the groups, after the time intervals of 7, 15, and 30 days, for the nine conditions evaluated

Condition Time/Days	Groups			*p* -Value *
	Control	Sealer	Experimental	
Inflammatory infiltrate				
7	1.00	1.20	1.40	0.725
15	0.20	0.40	1.00	0.067
30	0.00	0.00	0.40	0.285
Edema				
7	1.40	1.60	1.60	0.999
15	0.00 ^A^	1.00 ^B^	0.80 ^B^	0.006
30	0.00	0.00	0.40	0.285
Necrosis				
7	0.00	0.00	0.00	1.000
15	0.00	0.00	0.00	1.000
30	0.00	0.00	0.00	1.000
Granulation tissue				
7	0.00	0.40	0.40	0.450
15	0.00	0.00	0.00	1.000
30	0.00	0.00	0.00	1.000
Blood vessel				
7	2.55 ^A^	2.00 ^AB^	1.66 ^B^	0.020
15	2.44 ^AB^	1.44 ^A^	2.55 ^B^	0.022
30	2.55 ^A^	3.11 ^AB^	3.66 ^B^	0.002
Multinucleated giant cells				
7	4.14 ^A^	7.20 ^B^	5.00 ^AB^	0.001
15	2.00 ^A^	5.75 ^AB^	6.28 ^B^	0.001
30	2.00 ^A^	5.20 ^B^	2.00 ^A^	0.001
Young fibroblasts				
7	4.11 ^A^	4.55 ^AB^	5.55 ^B^	0.001
15	2.88 ^A^	3.44 ^AB^	4.00 ^B^	0.002
30	2.55 ^AB^	2.11 ^A^	3.88 ^B^	0.001
Fibrocytes				
7	1.77 ^A^	0.66 ^AB^	0.44 ^B^	0.004
15	0.44 ^AB^	0.33 ^A^	1.11 ^B^	0.034
30	1.11	0.66	0.55	0.115
Collagen				
7	2.00	1.80	1.57	0.230
15	2.75	2.40	2.00	0.090
30	3.00	3.00	2.66	0.285

a For each sample of the study, five representative sections of the histological condition of the tissue were analyzed.

* *p* -Value indicates nonparametric Kruskal–Wallis test, followed by Dunn's multiple comparisons test.

^A^ or ^B^ Means followed by the same single letter do not express statistically significant difference ( *p* > 0.05).

^AB^ Means followed by different letters express statistically significant difference ( *p* < 0.05).
